# Cell-Based Therapies for Rotator Cuff Injuries: An Updated Review of the Literature

**DOI:** 10.3390/ijms25063139

**Published:** 2024-03-08

**Authors:** Nicholas Hooper, Anuj Marathe, Nitin B. Jain, Prathap Jayaram

**Affiliations:** 1 Department of Physical Medicine and Rehabilitation, Emory University, Atlanta, GA 30322, USA; 2 Department of Physical Medicine Rehab and Orthopedics, University of Michigan at Ann Arbor, Ann Arbor, MI 48109, USA; jainnb@med.umich.edu; 3 Department of Orthopedics, Physical Medicine and Rehabilitation, Emory University, Atlanta, GA 30322, USA

**Keywords:** orthobiologics, rotator cuff tears, mesenchymal stem cells

## Abstract

This review focuses on non-surgical treatment options for rotator cuff injuries and highlights the potential of mesenchymal stem cells (MSCs) as a potential regenerative approach. MSCs, sourced from various tissues like bone marrow and adipose tissue, exhibit promising mechanisms in vitro, influencing tendon-related gene expression and microenvironment modulation. Animal studies support this, showcasing MSCs’ ability to reduce inflammation, improve tissue remodeling, and enhance repaired tendon strength. Human trials, while varied and limited, suggest that MSCs might lower retear rates and enhance post-repair outcomes, but randomized controlled trials yield mixed results, emphasizing the necessity for standardized investigations. Ultimately, while cell-based therapies demonstrate an excellent safety profile, more rigorous clinical trials are necessary to determine their efficacy in improving patient outcomes and achieving lasting structural changes in rotator cuff injuries.

## 1. Introduction

Shoulder pain is the third-most-common musculoskeletal complaint (behind back and knee pain) in the United States [1]. The prevalence of shoulder pain ranges from 14 to 34% [2,3,4,5,6,7] each year; about 1% of the population who are 45 years and older present with shoulder pain to primary care settings [8]. In the United States, the direct healthcare expenses attributable to shoulder disorders was estimated to be USD 7 billion in 2000 [9], and rotator cuff tears are considered one of the most expensive diseases treated in American hospitals [1]. Rotator cuff disorders are the underlying problems in 65–70% of patients with shoulder pain [10,11]. Despite this enormous public health impact, there are no disease-modifying treatments for rotator cuff tears.

The major symptomatic manifestations of rotator cuff tears include chronic shoulder pain, impaired mobility, and functional impairments. These arise due to progressive pathological remodeling of the tendon, leading to increased fibroblast cellularity, neovascularity, thinning/loss of collagen matrix, and fatty infiltration [12]. Rotator cuff tears can be treated non-operatively and operatively. The current non-surgical standard-of-care therapies such as physical therapy address biomechanical and functional deficits but do not regenerate the underlying structural tendon tear. In addition, non-steroidal anti-inflammatory drugs (NSAIDs), modalities (acupuncture, iontophoresis, etc.), and glucocorticoids injections provide symptomatic relief but do not prevent the progression of disease. Moreover, the rotator cuff tear size, muscle atrophy, and fatty infiltration may progress over 5 to 10 years with non-operative treatments [13,14]. The current guidelines for pharmacological therapeutic strategies that have been adopted by many professional organizations are largely focused on symptom relief in partial-thickness rotator cuff tears and do not offer disease-modifying benefits [14]. Alternative injections such as hyaluronic acid have limited evidence to support their use, and platelet-rich plasma has limited evidence that does not support routine use for treatment of rotator cuff tears [14]. Surgical treatments such as rotator cuff repair are also aimed at either debriding the tendon or anchoring a torn tendon back to the humeral head and do not alter the underlying tendon biology. Moreover, incomplete or failed tendon healing occurs in 20–25% of patients [14]. Thus, current treatments for rotator cuff tears are sub-optimal, and there is a significant need for disease-modifying therapies (DMTs).

Given the overall frequency of shoulder pain and rotator cuff tears, further treatment modalities are needed to aid with healing. Emerging regenerative options are based upon repurposing mesenchymal stem cells (MSCs) to directly treat existing tears in muscle fibers or augment surgical treatment options in cases of full-thickness tears. The purpose of this review is to provide a brief overview of MSCs and an update of the current literature regarding their clinical applications in treating rotator cuff tears.

## 2. Methods

**Figure 1:**
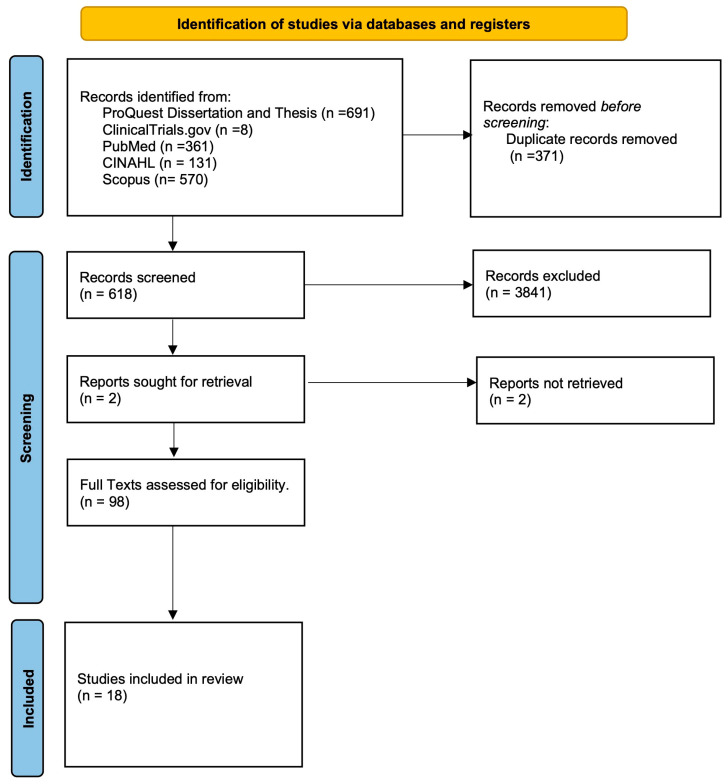
Flow chart of included studies.

Given the overall paucity of human controlled trials regarding the use of MSCs for rotator cuff pathology, the decision was made to pursue a scoping review. The aim of this study was two-fold: (1) to synthesize the current basic science of MSCs, understand the different subtypes of MSCs, and the current in vivo research of the use of MSCs for rotator cuff tears (RCTs), and (2) to evaluate the current literature regarding the use of MSCs for RCTs in humans. A detailed literature search (September 2023 to December 2023) in seven databases (PubMed (NLM); CINAHL; Scopus (Elsevier); ClinicalTrials.gov; and Proquest Dissertation and Thesis) in order to evaluate the evidence base for MSCs for RCTs (see Figure 1). For the purpose of this review, MSCs were defined as nonhematopoietic multipotent cells, which are capable of differentiating into a variety of cells of mesenchymal lineage [15], which could include tissue derived from almost all organs, including bones, adipose tissue, etc. Randomized control trials, as well as cohort studies and case series, were considered for inclusion.

## 3. Basic Science of Mesenchymal Stem Cells

MSCs are defined as nonhematopoietic multipotent cells, which are capable of differentiating into a variety of cells of mesenchymal lineage [15]. It is believed that they can be derived from the connective tissue of almost all organs, including bones, adipose tissue, dental pulp, as well as isolated from the human placenta, umbilical cord, and various fetal tissues [16]. The minimal criteria for identifying MSCs, as defined by the International Society for Cellular Therapy (ISCT), require the following: (1) must be plastic-adherent when maintained in standard culture conditions, (2) must express CD73, CD90, and CD105, and lack expression of CD11b, CD14, CD19, CD34, CD45, CD79a and HLA-DR surface molecules, and (3) be able to differentiate into osteoblasts, adipocytes, and chondroblasts in vitro [17].

Although initial studies theorized that MSCs repaired tissues through the differentiation and engraftment into injuries’ tissues, more recent research has shown that MSCs are able to mediate tissue repair, but they have only transient engraftment into the injured issues [16,18]. Recent studies suggest that MSCs’ therapeutic effects are mediated through the release of paracrine factors, mitochondrial transfer, and extracellular vesicle secretion [16,18,19]. MSCs produce an abundance of paracrine factors, including cytokines, chemokines, growth factors, and microRNA. Caplan and colleagues proposed that within local injury, MSCs actively participate in the suppression of local immune reactions within local tissues, as well as wound repair, tissue regeneration, and angiogenesis [20,21]. Furthermore, research has shown that MSCs can mediate the stimulation of the recruitment, proliferation, and differentiation of tissue-specific cells [21,22,23] and attenuate the oxidative stress response [21,24].

Although the ISCT definition states that MSCs must be able to differentiate into osteoblasts, adipocytes, and chondroblasts in vitro, some studies have shown that under appropriate conditions, MSCs can differentiate into other tissues, like tendon, skeletal muscle, myocardium, and smooth muscle [21,25,26]. Although MSCs can be harvested from a variety of tissues, many of these cells share similar characteristics. Research has shown some differences between these cells, which may, however, potentially lead to differences in differentiation propensity. For example, the global miRNA expression profile of MSCs varies according to the tissue of origin, which may affect cellular properties, such as proliferation, differentiation, and paracrine activities [27]. As such, it is important to understand the different MSCs’ harvesting sites/subtypes.

### 3.1. Mesenchymal Stem Cell Subtypes

MSCs can be harvested from multiple tissue sources of mesenchymal origin, including the placenta, umbilical cords, adipose tissue, bone marrow, as well as other tissues. Although multiple potential sources exist, the most commonly utilized adult sources are bone marrow and adipose tissue in orthopedics [28]. This is in part due to the ease at which these tissues are obtained, but also due to the success that these tissues have shown in producing a large number of MSCs and paracrine effects [28,29,30]. For the purpose of this review, we will further explore bone-marrow-derived MSCs, adipose-derived MSCs, umbilical-cord-derived MSCs, muscle, and peripheral blood.

#### 3.1.1. Bone-Marrow-Derived MSCs

Bone-marrow-derived MSCs (BM-MSCs) were the first MSCs identified and, thus, have been the most extensively studied, both in vitro and for their therapeutic properties [31,32]. BM-MSCs have been shown to comprise 0.001% to 0.01% of total marrow mononuclear cells [21,33,34]. As with all MSCs, BM-MSCs are thought to exert therapeutic effects through their ability to regulate cell proliferation/differentiation, ability to secrete trophic factors, and immunomodulatory activity. However, research has shown specific differences in BM-MSCs compared to other subtypes. Immunologically, BM-MSCs have been shown to strongly express CD49f, PODXL, CD 106, and cytochrome p450 and not express or minimally express CD54 and CD34, as compared to other MSCs [21,35,36]. In addition, some studies have demonstrated that BM-MSCs are more prone to osteogenic differentiation than other MSCs [21,35,37]. Additionally, some in vitro studies have found that some MSCs have decreased chondrogenic differentiation potential when compared to BM-MSCs [21,36,38].

As previously stated, this was the first stem cell discovered, and as such, it has been extensively investigated as a potential therapy for a wide variety of conditions, including, but not limited to, cardiovascular, neurological, orthopedic, oncologic, rheumatologic, and gastrological diseases.

#### 3.1.2. Adipose Tissue MSCs

Adipose tissue MSCs (AT-MSCs) have been also extensively studied due to their advantageous ability to be conveniently sourced as subcutaneous AT, which is abundantly found throughout the body. Unlike BM-MSCs, it is estimated that approximately 98–100% of cells obtained through AT are viable [39,40]. Thus, when compared to BM MASCs, AT-MSCs contain a 500-fold greater number of MSCs when isolated from an equivalent amount of adipose tissue [21,41]. MSCs can be harvest by either enzymatically digesting adipose tissue to yield a stromal vascular fraction (SVF) or through mechanical breakdown to yield micro-fragmented adipose tissue (MFAT). Studies have shown that MFAT contains higher concentrations of AT-MSCs when compared to SVF making it an ideal choice in clinical applications, especially given its comparative ease of accessibility [42,43]. However, one limitation of AT-MSCs is that certain donor characteristics, like age, can affect the ability of AT-MSCs to expand and differentiate, notably in the chondrogenic and osteogenic lineages [21,40,44,45]. However, these effects have not been clinically verified.

#### 3.1.3. Umbilical Cord Blood MSCs

Umbilical cord blood MSCs (UCB-MSCs) are considered an abundant source of mesenchymal stem cells. Since the MSCs derived from UCB are typically discarded at birth, some consider this a less expensive and the least invasive method of collecting MSCs compared to their adult source counterparts [46,47]. Another potential advantage of UCB is that, due to their immaturity, UCB-MSCs have been shown to be less immunogenic. In addition, they have been found to have a similar doubling time when compared to BM-MSCs [39,46,47,48]. Lastly, research has shown that they may have the highest expansion potential among all subtypes of MSCs [47].

#### 3.1.4. Muscle-Derived MSCs

As with other MSCs, muscle-derived MSCs (M-MSCs) are able to differentiate into multiple mesenchymal tissues, like myogenic, chondrogenic, and osteogenic linages. Of note, M-MSCs are committed to a myogenic lineage, while satellite cells are capable of multi-lineage differentiation [49]. Satellite cells are mononuclear cells that surround each muscle fiber and the plasma membrane of the fiber. These are thought to be the main cell type responsible for skeletal muscle regeneration. Studies have shown multiple potential applications for M-MSCs, including augmenting muscle healing following injury, both skeletal and cardiac, the promotion of peripheral nerve regeneration, and the promotion of vascular regeneration [39,50,51].

#### 3.1.5. Peripheral Blood MSCs

Peripheral blood progenitor cells have been shown to be mobilized through the use of filgrastim, a granulocyte-CSF [48,52]. An advantage of using mobilized peripheral blood MSCs (PB-MSCs) is the ease at which they can be accessed and obtained. They share the same ability to differentiate into mesenchymal lineages as other subtypes of MSCs; however, studies have shown that the doubling time of PB-MSCs is almost 95 h, which is longer than most other sources [48,53]. Lastly, another disadvantage of this subtype is that their capacity to differentiate into bone and chondral lineages has been shown to be lower than BM-MSCs.

## 4. MSCs for Rotator Cuff Injury

### 4.1. In Vitro Data

Tendon disruption seen in rotator cuff tears (RCTs) leads to decreased muscle fibers and mass, which subsequently leads to increased fat content [54,55]. That being said, RCTs are thought to induce fatty infiltration, and classification systems have been developed to quantify the severity of rotator cuff tears based on the degree of fatty infiltration [56]. It has been shown that surgical rotator cuff repairs have lower surgical success rates in patients with a more advanced Goutallier stage [55,57]. It is believed that the use of MSCs for tendinopathy reduces the inflammatory environment, shifting to a more reparative environment [58,59].

In vitro culture studies have attempted to better understand the mechanisms by which MSCs can aid in the repair of tendinopathies like RCTs. One study found that crosstalk between tendon cells and MSCs led to an upregulation of tendon-related genes, like scelraxis and tenomodulin, as well as tendon ECM markers, like type 1 collagen and decorin [59,60,61]. Another theory is that paracrine factors play a role in MSCs, supporting tendon cells. Sevivas and colleagues found that pre-conditioning tendon cells in vitro with the BM-MSCs’ secretome results in improved biomechanical performance when transferred to a rat model of rotator cuff tears [62]. Another potential mechanism by which MSCs treat RTC is through the generation of tendons like tissue. In one in vitro study, researchers were able to culture BMMSCs in fibrin gels and spontaneously generate collagen fibrils similar to embryonic tendons [59,63]. The rationale by which this occurs in vitro is due to TGF-B3 signaling.

Further studies have examined the potential cellular mechanisms by which AT-MSCs are able to improve tendon healing. AT-MSCs may also use a cellular crosstalk mechanisms in order in upregulate tendon-related genes [64,65]. Furthermore, in co-cultures with AT-MSCs and tendon explants, it was found that the collagenolytic activity of matrix metalloproteinase (MMPs) was increased. In addition to fastened extracellular matrix remodeling, the same study by Costa-Almeida and colleagues found an accelerated deposition of type 1 college and increased ratio of type 1 to type 3 collagen [66]. Thus, MSCs may play a role in shifting the microenvironment to induce repair and reduce fibrotic healing. Altogether, in vitro studies support the potential therapeutic effect for MSCs though multiple different mechanisms that lead to modulation of the microenvironment.

### 4.2. Clinical Applications of Mesenchymal Stem Cells in Rotator Cuff Disease

#### 4.2.1. In Vivo Studies

RCTs can manifest in varying severity and with varying levels of fibrosis and fatty infiltration. In a study conducted by Mora et al. in 2014, a rat model of acute supraspinatus tear followed by repair was used to investigate the effects of AT-MSCs [67]. Their findings a revealed notable reduction in acute inflammation, edema, and a decreased presence of neutrophils in histology. Similarly, Chen et al. (2015) conducted a study using human-adipose-derived MSCs in a rat model of RCT [68]. They observed improved fiber arrangement and tendon organization, as well as reduced inflammation. These findings suggest that AT-MSCs can help mitigate the initial inflammatory response following RCT. They also show promise in chronic disease, as seen in a study by Gunmucio et al. (2016), who investigated stromal vascular stem cell treatment in conjunction with surgical repair in a rat model of chronic RCT [69]. Their results showed a significant reduction in muscle fibrosis, up to 40% when compared to repair alone.

Furthermore, MSCs have shown promise in enhancing functional outcomes following RCT repair. In a rabbit model of chronic RCT, researchers demonstrated that adipose-derived MSC exosomes, when injected after surgical repair, led to a significantly higher tendon load to failure, increased muscular stiffness, and improved tendon stress tolerance compared to surgical repair alone [70]. This suggests that MSCs can play a pivotal role in augmenting the mechanical integrity and functional performance of repaired tendons. Shin (2020) utilized adipose-derived MSC cell sheets in a rat model to improve tensile strength, particularly at the enthesis following rotator cuff repair [71]. The nearly two-fold increase in tensile strength highlights the potential of MSC-based therapies to enhance the structural integrity of the repaired tendon, which is crucial for functional recovery. Finally, multiple studies have employed scaffold matrices populated with MSCs in rat models of RCT, resulting in increased tendon tensile strength [72,73].

While the precise mechanisms underlying these improvements remain unclear, it is believed that MSCs may modulate this process through a paracrine activity of suppressing pro-inflammatory cytokines, which can hinder tendon healing after injury, and by simultaneously promoting angiogenesis to improve cellular healing [74]. Nevertheless, these in vivo trials provided compelling evidence for the therapeutic potential of mesenchymal stem cells in addressing both the structural and functional aspects of tendon healing in RCTs. These studies suggest that MSCs can reduce inflammation, enhance tissue remodeling, and improve the mechanical properties of repaired tendons, which has prompted human clinical trials.

#### 4.2.2. Human Trials

Overall, our review found 18 case reports/series, RCTs, and case control series examining the effects of MSCs of rotator cuff tears (see Table 1) [75,76,77,78,79,80,81,82,83,84,85,86,87,88,89,90,91,92]. The most notable features of each study are summarized in Table 1. Of note, one of the included studies was a 2-year follow-up from the initial study. While human trials for MSC use in musculoskeletal applications have been on the rise in recent years, there continues to be a paucity of well-designed studies, especially randomized controlled trials, that examine the effects of MSCs of RCT. There is a lot of variance in target populations, MSC type and application, and follow-up time frames. We aim to synthesize the available evidence below.

Of the 18 studies reviewed, 12 were either case series, case reports, or case-controlled studies. Five of the included non-randomized controlled studies examined BMAC, while the other seven examined AT-MSCs/MFAT. In an earlier human-based study involving MSCs, Hernigou and colleagues showed, in their 2014 case-controlled study, that using bone-marrow-derived MSCs as an adjunct to surgical rotator cuff repair could help prevent retears in the future (as seen at 10 yr follow-up) and improve the quality of the repair [82]. Interestingly, they also found that the number of MSCs that were transplanted positively correlated with a patient’s tendon integrity. Similarly, a cohort study conducted by Kim et al. (2017) showed that using adipose-derived MSCs to augment surgical rotator cuff repair significantly decreased retear rates, as seen on MRI [87]. However, they did not note any clinical differences in patients on follow-up. On the other hand, 10 of the 12 included studies found improvements in pain and/or functional outcome scores. In fact, Jo et al. (2018) found improvements in pain of up to 80%, with arthroscopic evaluation demonstrating near-full healing of the tear defect [85,86]. The positive results from these types of studies helped open the door to future randomized control trials. Currently, there are nine registered trials on clinical trials.gov classified as “recruiting, unknown status, active, or completed” (see Table 2).

#### 4.2.3. Human Randomized Control Trials

Of the 18 studies, our review found that only 5 were randomized control trials. Two of these RCTs examined BMAC, while the other three studies examined AT-MSCs/MFAT. One of the most recently published trials was by Cole et al. (2023) in 2023, where they compared arthroscopic rotator cuff repair alone versus repair augmented with concentrated bone marrow aspirate in 91 patients [79]. They found that patient-reported pain and function outcomes were not statistically different between groups. However, they noted significantly lower retear rates (18% vs. 57%; *p* < 0.001) in the augmented repair group based on Sugaya classification on one-year MRI scans. In contrast, Randelli et al. (2022) conducted a similar trial but, this time, using adipose-derived MSCs in the form of microfragmented adipose tissue to augment arthroscopic repair [92]. They followed patients for a total of 24 months and found that at 6 months, the augmented repair group had statistically significant improvements over the control group in patient-reported pain and function. Interestingly, these differences were not seen at any other follow-up point. These studies suggest that MSCs can certainly help augment the healing process when used in conjunction with arthroscopic repair, but their clinical significance for patient-reported metrics needs to be further investigated.

Mixed results were noted in studies that investigated non-surgical MSC injections as well. Centeno et al. (2020) compared bone marrow concentrate plus PRP injection to exercise therapy in partial-thickness supraspinatus tear and found significant improvements in pain and function outcomes at 12 months [76]. Similarly, Hurd et al. (2020) compared injections of adipose-derived MSCs versus steroids in partial-thickness rotator cuff tears who had failed treatment with physical therapy [84]. They found statistically significant improvements in pain and function outcomes at 12 months [84]. However, this was in a small sample size of 16 patients, as their published results were pilot data, and the study is still ongoing. On the other hand, Chun et al. (2022) compared injections with adipose-derived MSC plus fibrin glue, fibrin glue only, and saline only for the treatment of partial-thickness supraspinatus tears and found no significant differences in pain or functional patient-reported outcomes [78].

While some studies [76,84,92] suggest there may be short-term clinical benefit for patients, larger, more rigorous trials will be required to fully elucidate the extent of the clinical benefit that can be expected from this orthobiologic treatment. For instance, future studies can include comparisons between different formulations of MSCs (bone marrow vs. adipose, etc.), a comparison of treatment efficacy in full-thickness vs. partial-thickness tears, and long-term follow-ups.

## 5. Future Directions with MFAT

A major challenge in developing drug-modifying therapy is the necessity to modulate several dysregulated pathways that impact pain, intra- and peri-tendinous inflammation, and structural tendon loss. One approach to achieve such disease modification is through orthobiologic agents, such as MSCs, that are formulated with specific tendon stem/progenitor cells that can potentially reduce tendon inflammation and pain, enhance overall function, and repair tendon tear loss [93].

Overall, there are limited RCTs examining the effect of MSCs on rotator cuff tears. To date, there is only one double-blinded randomized control trial that shows the beneficial effects of MSCs at 6 months [80]; however, this is in conjunction with arthroscopic repair of large rotator cuff tears compared to arthroscopic repair alone and does not include a non-operative injection arm of MSCs alone. In addition, only a few unblinded prospective trials evaluating MSC-based therapies for rotator cuff tears exist and demonstrate an excellent safety profile; however, translating the results of these trials into clinical practice is challenging due to key limitations, including the following: (1) heterogeneity of MSC formulations, (2) lack of standardization for dosing and/or administration frequency, (3) lack of trials utilizing endpoints that assess disease-modifying properties [85,86,87]. Moreover, no trial has comprehensively defined a formulation that is reproducible with specific biological properties in patients with partial-thickness rotator cuff tears. Given this, it is important that future clinical trials focus on standardizing formulations and developing standardized administration frequencies in order to properly assess the outcomes of MSCs when used for rotator cuff tears.

## 6. Conclusions

Cell-based therapy has certainly been shown to be safe in human use when derived from both bone marrow and adipose tissues. This review has clearly shown that a fair number of studies have been conducted to demonstrate safety; however, more well-designed robust clinical trials need to be carried out to assess its efficacy in patient outcomes and determine mechanistically if structural modification is a resulting long-term outcome.

## Figures and Tables

**Table 1:** Current published studies examining MSCs for rotator cuff tears. Included are the study name, study design, pathology, number of total participants, harvesting site, outcomes, and results.

Study	Study Design	Pathology	Number of Patients	Type of MSCs	Adjunctive Treatment Modalities	Outcome	Follow-Up	Results
Centeno, et al., 2015 [75]	Case Series	GH OA and/or partial/full rotator cuff tears	115 (81 RCT and 34 OA)	Bone Marrow Concentrate (BMAC)	NA	DASH and NPS	3 months	Significant improvement of DASH and NPS scores
Centeno, et al., 2020 [76]	Randomized Controlled Crossover trial	Chronic partial or full thickness non-retracted rotator cuff tears	25	Bone Marrow Concentrate (BMAC)	Control-Exercise Therapy	Primary-DASH Secondary-NPS, SANE	1, 3, 6, 12, 24 months	Significant differences for BMAC over exercise group at 3 and 6 months for pain, and for function and reported improvement
Cherian et al., 2019 [77]	Case Report	Chronic rotator Cuff Tear	1	Microfragmented adipose tissues (MFAT)	NA	NRS, WUSPI, BPI-17, PGIC	1, 2, 3, 6, 12 months	Complete pain relief in all outcome measure from 1–12 months
Chun et al., 2022 [78]	Randomized control trial	Partial tear of supraspinatus tendon	24 across 3 groups	Microfragmented adipose tissues (MFAT)	Control-Normal Saline (NS) Group 2-Fibrin glue and NS	Primary: VAS at 3 months Other: VAS, ASES, MRI Imaging	6 weeks, 3, 6,12 months	No significant difference found in pain scores 3 months post injection
Cole et al., 2023 [79]	Randomized control trial	Supraspinatus tendon tears undergoing rotator cuff repair (RCR)	91	Bone Marrow Concentrate (BMAC)	BMAC + RCR versus NS + RCR	PROMs, ASES, SANE, Veterans RAND, MRI	6, 12,24 months	Functional outcomes significantly improved in both groups. The control group had significantly greater evidence of rotator cuff retear at 1 year MRI
Ellera Gomes et al., 2011 [80]	Case series	Full thickness rotator cuff tear	14	Bone Marrow Concentrate (BMAC)	Rotator Cuff Repair Surgery	UCLA, MRI	12 months	Improved functional score. Tendon integrity in all cases at 12 months
Ferrell, JL et al., 2023 [81]	Case Report	Full thickness Supraspinatus tear	1	Microfragmented adipose tissues (MFAT)	NA	DASH, MRI	1, 6, 8 months	Improved DASH Scores
Hernigou, et al., 2014 [82]	Case Control Study	Full thickness supraspinatus tears	90	Bone Marrow Concentrate (BMAC)	BMAC + RCR versus RCR	Imaging findings on US (monthly) or MRI	US (monthly) or MRI at 3, 6, 12, 24 months as well as MRI At 10 years	BMAC improved the rate of healing at 6 months and decreases retear rate at 10 years
Hogaboom et al., 2021 [83]	Pre-post clinical trial	Chronic Rotator cuff Tear	10	Microfragmented adipose tissues (MFAT)	NA	NRS, WUSPI, BPI-17, PGIC	6 and 12 months	WUSPI, NRS, and BPI-I7 scores were significantly lower 6 and 12 months post-procedure
Hurd, et al., 2023 [84]	Randomized Control Trial	Partial thickness rotator cuff tears	20	adipose tissue-derived mesenchymal stem cells (AT-MSCs)	Control- Corticosteroids	Primary- Adverse outcomes Secondary-ASES, RAND, VAS, MRI	Assessments at 3, 6, 9, 12, 24, 32, 40, and 52 weeks. MRI at 24 and 52 weeks	No adverse outcomes were reported 12 months post treatments. Those in intervention group showed significantly higher mean ASES total scores at W24 and W52 post treatment
Jo et al., 2018 (and follow up paper in 2020) [85,86]	Case Series	Partial Thickness tears	18	adipose tissue-derived mesenchymal stem cells (AT-MSCs)	NA	SPADI, Adverse events, Constant score, VAS, and MRI	1, 3, 6 months and 2 year study follow up	No serious adverse events through 2 years. SPADI and CS significantly improved in mid- and high-dose groups. Shoulder pain recuded by 90% at 2 years by the mid and high dose groups.
Kim, et al., 2017 [87]	Cohort Study	Full thickness rotator cuff tears	70	adipose tissue-derived mesenchymal stem cells (AT-MSCs)	AT-MSCs+ RCR versus RCR	VAS, ROM, UCLA, MRI	12 months	No functional difference. MRI with significantly higher retear rate in just RCR group (28.5 vs. 14.3)
Kim et al., 2017 [88]	Case Series	Partial Thickness rotator cuff tears	12	Bone Marrow Concentrate (BMAC)	BMAC + PRP	ASES, VAS, US	3 weeks, 3 months	Significant improvement in pain (VAS) and ASES scores after 3 months.
Kim et al., 2018 [89]	Case control study	Partial Thickness rotator cuff tears	24	Bone Marrow Concentrate (BMAC)	BMAC + PRP versus rehabilitation alone control	ASES, VAS, US	3 weeks, 3 months	Significant improvement in pain at 3 months for experimental versus control group. The change in the tear size did not differ significantly between groups
Marathe, et al., 2021 [90]	Case Study	Partial thickness Supraspinatus tear	1	Microfragmented adipose tissues (MFAT)	MFAT + PRP	VAS, ROM, US	14 and 28 weeks	Significant improvement in pain and mobility at 28 weeks. Resolution of the tear on US
Striano, et al., 2018 [91]	Case Series	Chronic Rotator Cuff Tear	18	Microfragmented adipose tissues (MFAT)	NA	NPS, ASES	1 and 5 weeks, 3, 6, and 12 months	Significant improvement in pain and ASES scores at all timepoints
Randelli, et al., 2022 [92]	Randomized control trial	Rotator Cuff Tears	44	Microfragmented adipose tissues (MFAT)	MFAT + RCR versus RCR	Constant Murley Score, ASES, VAS, Strength	3, 6, 12, 18, 24 months	Significant difference favoring experimental group of CMS scores at 6 months. No significant differences in rerupture rate or adverse events between groups

**Table 2:** This table represents currently registered studies on clinicaltrails.gov when searching for “Rotator Cuff Tear” and “Stem Cells”. Only studies classified as recruiting, unknown status, active, or completed were included in the table. Any study classified as withdrawn or suspended was not included. The quality of these studies was not evaluated as they are currently ongoing.

Indication	Study ID	Location	Intervention	Control	Number of Patients	Source of MSCs
MSCs in reconstruction Surgery of Supraspinatus Muscle Lesions	NCT03068988	Hospital Znojmo, Czechia	Single Injection of BM-MSCs intra-op	Surgical Repair without MSCs	50	BM-MSCs
Use of MSCs in patients with supraspinatus partial thickness tear	NCT02298023	Seoul National University Hospital, South Korea	Single Injection of AT-MSCS	Saline Injection	24	AT-MSCs
Use of MSCs in patients undergoing Arthroscopic Rotator Cuff Repair	NCT02484950	Rush University Medical Center	Single Injection of BM-MSCs intra-op	Surgical Repair without MSCs	100	BM-MSCs
AT-MSC for partial Thickness rotator cuff tear	NCT04077190	Fargo, North Dakota	Single Ultrasound guided Injection of AT-MSCs	Cortisone Injection	15	AT-MSCs
AT-MSCs for symptomatic partial thickness rotator cuff tears	NCT03752827	Mutli-center	Single Ultrasound guided Injection of AT-MSCs	Corticosteroid	246	AT-MSCs
BMAC for non-retracted supraspinatus tendon tear	NCT01788683	Broomfield, Colorado	Single injection of BMAC under imaging guidance	Exercise Therapy	51	BM-MSCs
Use of AT-MSCs on clinically diagnosed rotator cuff tear or lateral epicondylitis	NCT03279796	Zhejiang University, China	Single Injection of AT-MSCs	Betamethasone	200	AT-MSCs
Use of MSCs with reconstructive surgery in patient with complete supraspinatus tendon tears	NCT01687777	Hospital San Carlos, Spain	MSCs included within collagen type 1 membrane	Surgical repair with collagen type 1 membrane	10	Not specified
Use of MFAT in SCI patients with diagnosed rotator cuff disease	NCT03167138	Kessler Institute for Rehabilitation, New Jersey	Single Injection of micro-fragmented adipose tissue	None	10	AT-MSCs

## Data Availability

Data sharing not applicable. No new data were created or analyzed in this study. Data sharing is not applicable to this article.
